# Divergent Population Trends of Two Sympatric Auk Species in the Rapidly Warming Gulf of Maine

**DOI:** 10.1002/ece3.70495

**Published:** 2024-11-20

**Authors:** Sarah E. Durham, Sarah P. Saunders, Antony W. Diamond, Thomas V. Riecke, Heather L. Major

**Affiliations:** ^1^ Department of Biological Sciences University of New Brunswick Saint John New Brunswick Canada; ^2^ National Audubon Society New York New York USA; ^3^ Atlantic Laboratory for Avian Research University of New Brunswick Fredericton New Brunswick Canada; ^4^ Wildlife Biology Program University of Montana Missoula Montana USA

**Keywords:** climate change, integrated population model, long‐term monitoring, population dynamics, seabirds, southern range‐edge populations

## Abstract

Rapidly warming global temperatures are having a widespread influence on wildlife communities across taxa, with southern‐edge populations often experiencing the greatest negative impacts. However, sympatric species may exhibit divergent demographic responses due to differences in life history strategies and niche separation. We used integrated population models to estimate abundance, survival, and productivity for Atlantic Puffins and Razorbills nesting at the southern edge of their breeding range in the rapidly warming Gulf of Maine. We then conducted transient life table response experiments to understand the relative importance of demographic parameters in driving population dynamics. We found that the Atlantic Puffin population remained relatively stable over the 22‐year study period, whereas the Razorbill population increased substantially. Estimates of mean survival and productivity were similar between the study species but were at the lower range of values reported in the literature across their range. Despite similar estimates of mean productivity, interannual variation in this demographic rate was much higher in Puffins than Razorbills. Overall, adult survival was found to be the primary driver of population dynamics for both species yet shows evidence of long‐term decline in Puffins. For Razorbills, we found similar evidence of long‐term decline in first‐year survival. Overall, our findings suggest that these sympatric species may be responding differently to shared environmental conditions. Given the observed long‐term decrease in Puffin adult survival, future monitoring and conservation efforts for this species should be focused outside the breeding season in critical overwintering areas and migratory locations where adult mortality is typically concentrated. Similarly, given the observed long‐term decline in Razorbill first‐year survival, additional monitoring and tracking of chicks is warranted for this species to understand where immature individuals are going after they fledge from the colony.

## Graphical abstract

The Gulf of Maine is warming faster than much of the global ocean and represents the southern breeding range edge for Atlantic Puffins (*Fratercula arctica*) and Razorbills (*Alca torda*). To understand how the dynamics of these southern‐edge populations have changed over time, we used integrated population models to estimate abundance, survival, and productivity from 1998 to 2019. Overall, we found evidence of long‐term decline in adult survival for Puffins and first‐year survival for Razorbills suggesting that these southern‐edge populations may be experiencing demographic impacts due to rapid warming in the Gulf of Maine.

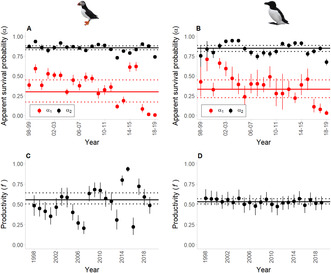

## 1 Introduction

Rapid and unprecedented global changes are having widespread and synergistic impacts on populations and communities (Jenouvrier 2013; Northrup et al. 2018). Specifically, in response to a warming climate, species are shifting their distributions (Parmesan and Yohe 2003), and populations at southern range edges often experience the greatest impacts (Jiguet et al. 2010; Hastings et al. 2020; Lewis et al. 2022). As a result, understanding the population dynamics of species residing at the southern edge of their breeding distribution provides important insights into how they may be responding to rapidly changing environmental conditions. Despite shared environmental conditions, sympatric species (i.e., species overlapping in their distributions) may exhibit divergent demographic responses due to differences in life history strategies and niche separation (Loreau and de Mazancourt 2008; Robinson, Dornelas, and Ojanguren 2013). As such, assessing interspecific demographic synchrony can enhance targeted management efforts.

Integrated population models (IPMs) are a relatively recent development that use data from the population level (e.g., population counts) in combination with data from the individual level (e.g., productivity and survival) in a unified modeling framework (Schaub and Abadi 2011; Plard et al. 2019). This approach allows for both simultaneous estimation of population abundance and demographic rates, and estimation of demographic parameters for which explicit data are unavailable (Besbeas et al. 2002; Schaub and Abadi 2011), such as juvenile survival and stage‐ or age‐specific population sizes in species with delayed maturity. Combining IPMs with recently developed transient life table response experiments (tLTRE) allows us to understand how specific demographic rates and the age/stage structure of the population may influence population dynamics (Koons, Arnold, and Schaub 2017). Comparing these analyses between sympatric species provides insight into (1) demographic responses to shared environmental conditions and (2) demographic synchrony within a community.

Two sympatric species, the Atlantic Puffin (*Fratercula arctica*, hereafter ATPU) and Razorbill (*Alca torda*, hereafter RAZO) overlap in their breeding distributions in the Gulf of Maine (GoM). Both ATPU and RAZO are cold‐water‐adapted seabirds with the GoM representing the southern edge of their breeding range (Lowther et al. 2020; Lavers, Hipfner, and Chapdelaine 2020). This area is warming faster than most of the global ocean (Pershing et al. 2015; Seidov, Mishonov, and Parsons 2021), with cascading effects on the marine ecosystem. Prolonged periods of abnormally high ocean temperatures (i.e., marine heatwaves) occurred in 2013 and 2016 in which sea surface temperatures were 1°C–3°C warmer than the 1982–2011 average (Mills et al. 2013). Warming waters have contributed to changes in the distribution and abundance of important prey species like Atlantic herring (*Clupea harengus*; hereafter “herring”), which have largely been replaced by lower‐quality species like silver hake (*Merluccius bilinearis*) in the diet of ATPU and RAZO since 2010 (Nye et al. 2009; Scopel et al. 2019; Depot et al. 2020). These shifts in prey have been linked to reduced productivity in both species (Kress, Shannon, and O'Neal 2016; Whidden 2016; Scopel et al. 2019), and adult survival in ATPU has been found to be positively correlated with herring landings and chick diet (Breton and Diamond 2014). Additionally, reductions in ATPU body size have been attributed to decreasing prey quality and increasing ocean temperature (Major et al. 2024).

Previous demographic analyses have been conducted for ATPU and RAZO in the GoM (Breton, Diamond, and Kress 2005, 2006a; Lavers et al. 2008), but those studies used shorter time‐series which did not encompass the prey regime shift and more recent marine heatwave events (Mills et al. 2013; Pershing et al. 2018). Therefore, in this study we use IPMs to jointly estimate annual survival, productivity, and population size for ATPU and RAZO breeding on Machias Seal Island (MSI) in the GoM from 1998 to 2019. We then compare annual survival and productivity estimates obtained from the IPM to those obtained from separate analyses of each individual dataset to evaluate model performance. Finally, we use post hoc tLTRE to determine the relative importance of demographic parameters in driving population dynamics. If the demographic responses and driver(s) of population dynamics are similar for both species, then management actions targeting one species will likely benefit both. However, if species respond differently to shared environmental conditions, management actions would need to be more tailored to species‐specific needs and recovery goals. Understanding the population dynamics of southern‐edge species may help to predict future responses in other parts of their range.

## 2 Methods

### 2.1 Demographic Data Collection

**FIGURE 1:**
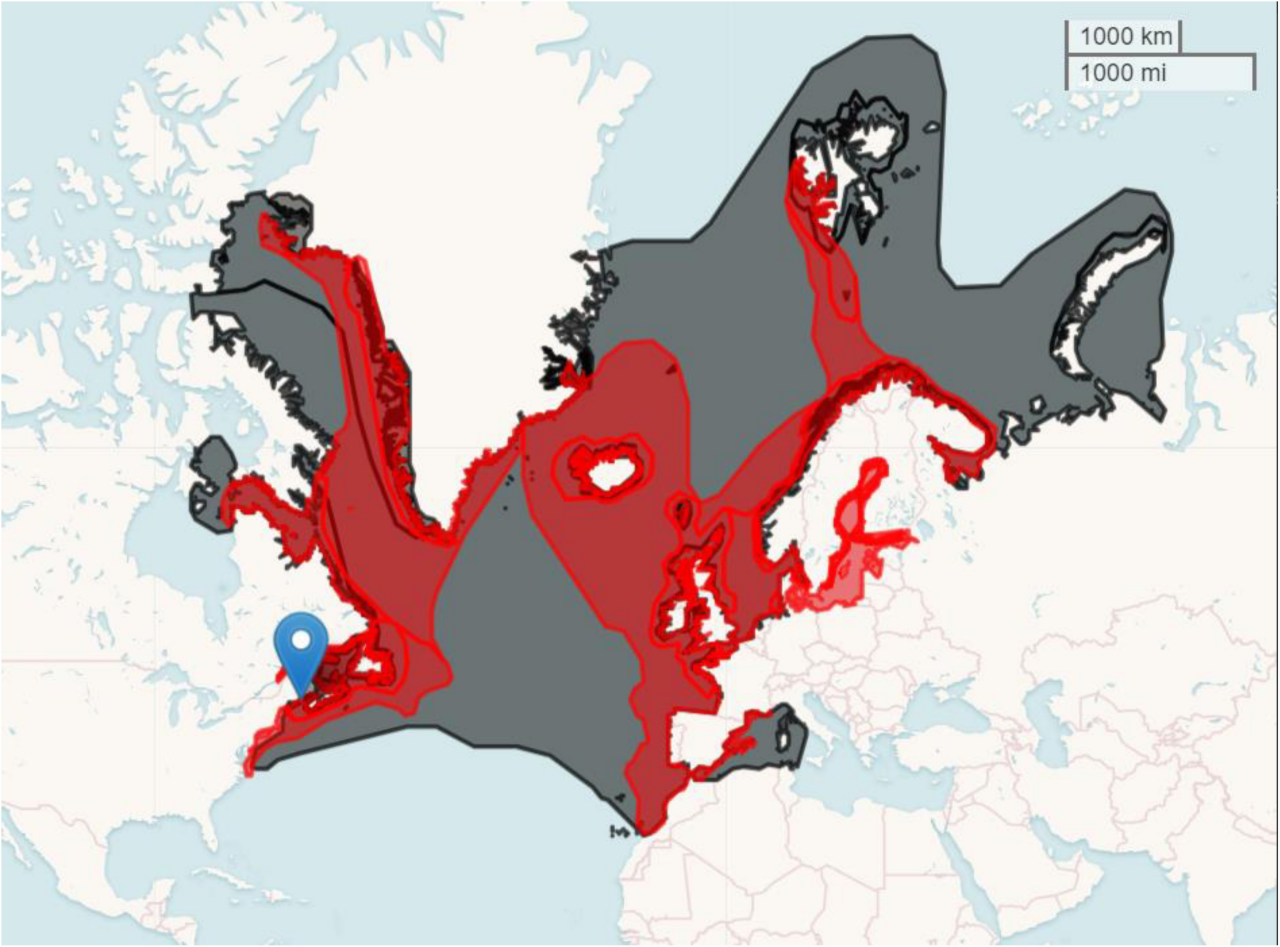
Global range map for ATPU (black; BirdLife International 2018) and RAZO (red; BirdLife International 2021a) with the location of Machias Seal Island (44°30′ N, 67°06′ W) indicated by blue marker.

Machias Seal Island (44°30′ N, 67°06′ W, hereafter MSI; Figure 1) is the largest breeding colony of ATPU and RAZO in the GoM with approximately 8600 and 3715 breeding pairs, respectively (Diamond 2021). Demographic data (breeding pair counts, capture‐mark‐recapture, and productivity) for each species were collected on MSI from 1998 to 2019 by the Atlantic Laboratory for Avian Research at the University of Brunswick (Diamond 2014, 2021).

#### 2.1.1 Breeding Pair Count Data Collection

**FIGURE A1:**
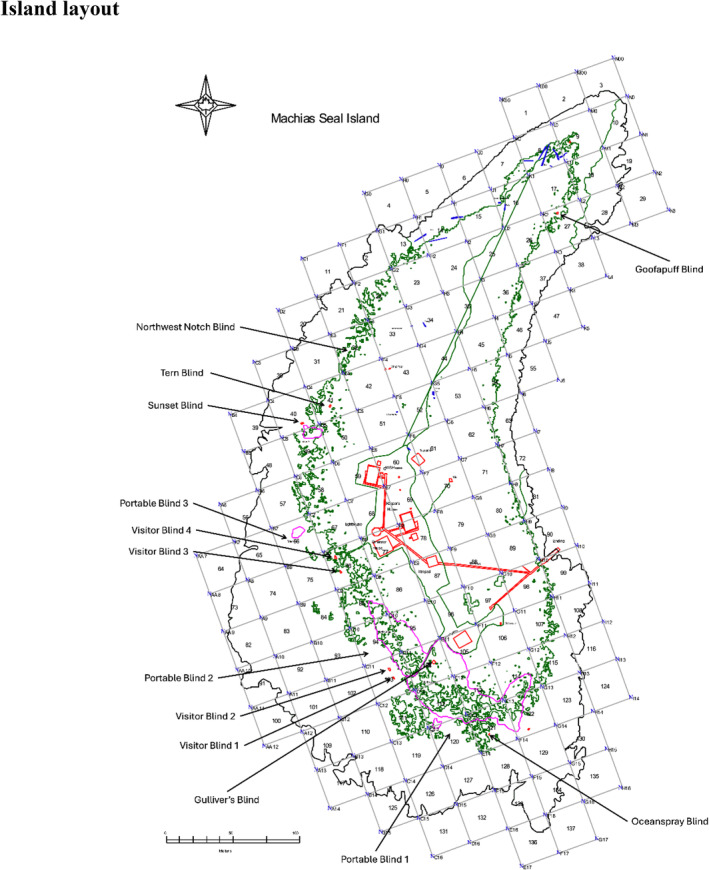
Map of Machias Seal Island showing 30 m grid squares and locations of portable and stationary (structured) research blinds.

**FIGURE A2:**
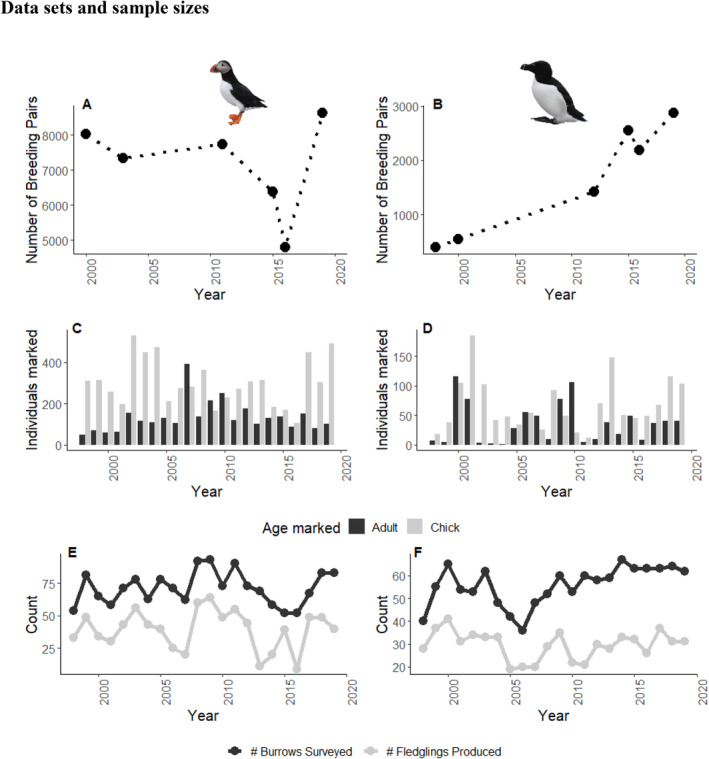
Data sets used for integrated population models and single data set analyses. Breeding pair count data show the number of breeding pairs counted in each of six survey years for (A) ATPU and (B) RAZO. Capture‐mark‐recapture data sets included the number of adults and chicks marked each year for (C) ATPU (total of 6669 chicks and 2943 adults) and (D) RAZO (total of 1479 chicks and 785 adults). Breeding success data sets included the number of burrows surveyed and the number of chicks fledged each year for (E) ATPU (total of 1566 burrows 862 fledglings), and (F) RAZO (total of 1227 burrows 651 fledglings).

Breeding pair counts came from systematic surveys of breeding burrows after peak lay but prior to peak hatch. This timing is optimal in that it minimizes the potential for undercounting by avoiding missing nest initiation attempts that had not yet begun, as well as minimizing the number of failed or already hatched nests. For each survey, 2 × 2 m quadrats were placed every 5 m along established gridline transects (defined by 30 m grid squares; Appendix 1: Figure A1) running southwest to northeast along the entire 8 ha island. The status of each burrow within the quadrats was recorded as either occupied (presence of egg or chick), unoccupied (empty), or unknown (contents unable to be determined). Assuming even distribution of breeding burrows, the number of occupied burrows per area surveyed (determined by multiplying the total number of quadrats by the quadrat area) was then extrapolated to the entire breeding area (i.e., appropriate breeding habitat) of the colony (approximately 20,000 sq. m) as a proxy for the total number of breeding pairs. From 1998 to 2019, a total of six surveys were conducted for both ATPU and RAZO (Appendix 1: Figure A2A,B). The extrapolated number of breeding pairs in each survey year were used to estimate total breeding population size.

#### 2.1.2 Capture‐Mark‐Recapture (CMR) Data Collection

**FIGURE A3:**
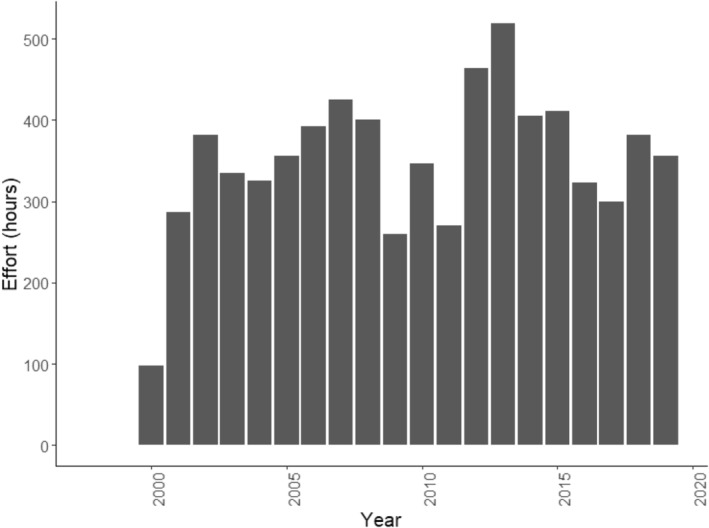
Capture‐mark‐recapture resighting and trapping effort (in hours; total = 7037.76) for ATPU and RAZO on MSI from 1998 to 2019.

Annual CMR data were collected by banding adults and chicks (i.e., known age individuals) with unique alpha‐numeric leg bands. Across the entire study period, there were a total of 6669 chicks and 2943 adults marked for ATPU and 1479 chicks and 785 adults marked for RAZO (Appendix 1: Figure A2C,D). Observers then attempted to resight as many individuals of both species as possible each breeding season from six stationary and two to three portable research blinds (Appendix 1: Figure A1) during multiple 3‐h stints (Appendix 1: Figure A3). Birds were also re‐encountered through physical recaptures at burrows and during trapping events. Unique band numbers observed during an encounter (recapture or resight) were recorded. Each breeding season after 1998 was considered a re‐encounter occasion, and these CMR data were used to estimate annual apparent survival probability from breeding season to breeding season.

#### 2.1.3 Productivity Data Collection

Annual breeding success data for both species (Appendix 1: Figure A2E,F) came from monitoring ~75–100 marked burrows each breeding season. Marked burrows were chosen based on accessibility to researchers and the location of the burrow within the colony (in order to obtain a representative sample from the entire breeding area of the colony). These burrows were checked at regular intervals for lay and hatch to ensure that the approximate age of the chick was known. To determine how many chicks fledged from active burrows (i.e., burrows with an egg), the burrows were regularly checked during the growth phase of the chicks up until fledge (see Diamond 2014 for specific details regarding productivity protocols). Chicks were assumed to be fledged when they were approximately 35 days old for ATPU (Harris and Wanless 2011) and 15 days old for RAZO (Lavers, Hipfner, and Chapdelaine 2020) and the burrow was empty on two weekly successive checks. The number of chicks fledged annually was considered the productivity data and used to estimate per‐capita fecundity.

### 2.2 Analysis—Integrated Population Model

**FIGURE A4:**
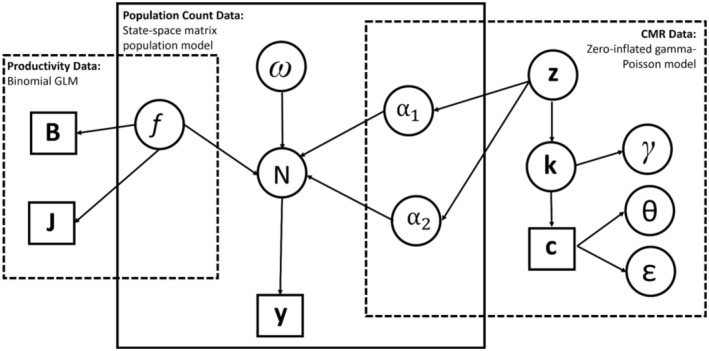
Direct acyclic graphical representation of the integrated population model for Atlantic Puffins and Razorbills. Observed data are represented by squares (*B* = number of monitored burrows, *J* = number of chicks fledged from monitored burrows, *y* = breeding pair count data, *c* = number of times each individual was observed during each breeding season) while estimated parameters are represented by circles (*f* = productivity, *ω* = immigration rate plus noise parameter, *N* = population size, $\alpha_{1}$ = first‐year survival, $\alpha_{2}$ = adult (after first year) survival, *z* = latent state (alive or dead), *k* = availability for detection, *γ* = probability of available for detection, *θ* overdispersion parameter defining individual heterogeneity in detection, and *ε* = mean expected number of encounters per individual). Arrows indicate dependencies among the observed data and parameters. Large boxes represent individual submodels for the different datasets. Parameters that are within multiple submodel boxes indicate that estimates are informed by multiple datasets.

To estimate apparent survival, productivity, and population size, we developed a female‐based IPM to jointly analyze the three data sets. Thus, the IPM consisted of three sub‐models: a state‐space matrix population model using the count data, a zero‐inflated gamma‐Poisson model using the CMR data, and a binomial GLM using the productivity data (as breeding pairs only rear one check to fledge each year; Appendix 1: Figure A4).

#### 2.2.1 Count Data Submodel

**FIGURE 2:**
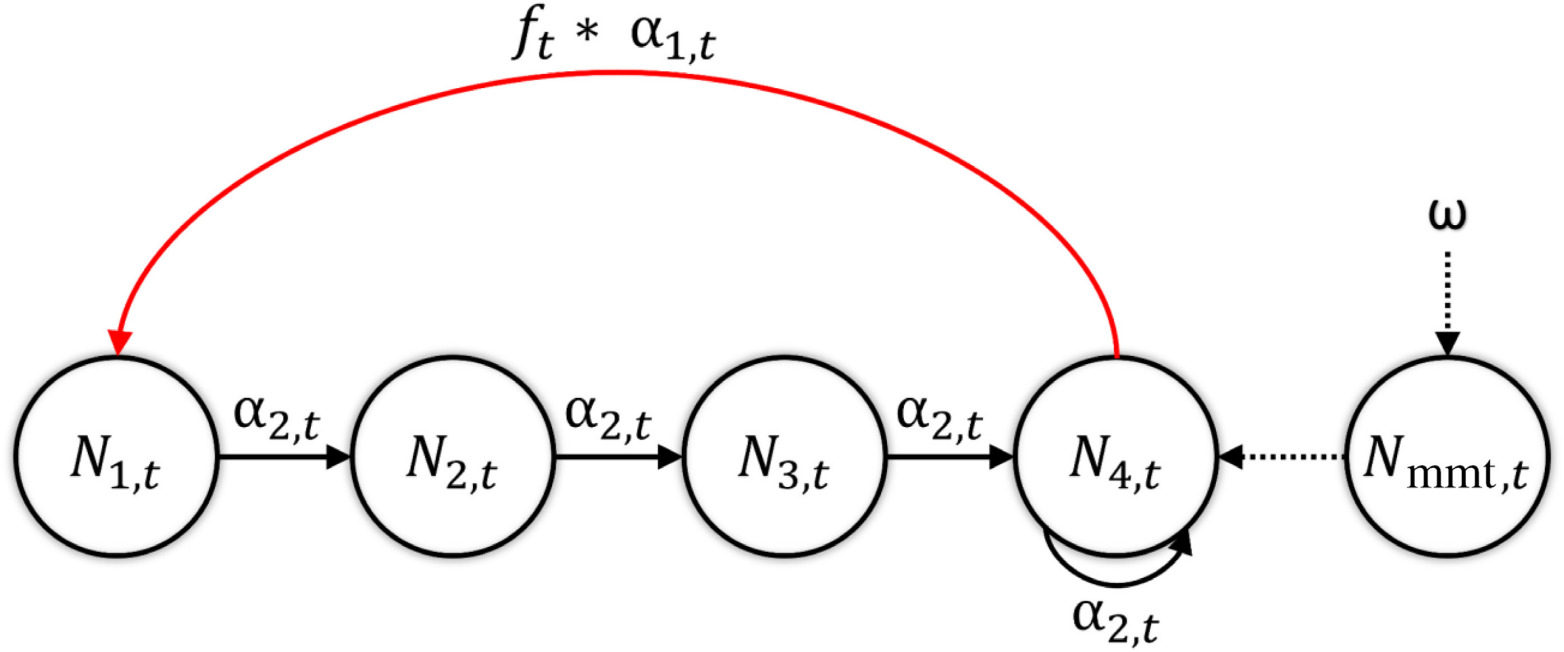
Life cycle graph for ATPU and RAZO representing a pre‐breeding census and female based model with five classes: 1st year ($N_{1,t}$), 2nd year ($N_{2,t}$), 3rd year ($N_{3,t}$), and 4th year and older individuals ($N_{4,t}$), and immigrants ($N_{\mathrm{imm}}$). The red arrow indicates the recruitment process with $\alpha_{1,t}$ representing first‐year survival and $\left(f_{t}\right)$ representing productivity. The black arrows indicate the survival process with $\alpha_{1,t}$ representing first‐year survival and $\alpha_{2,t}$ representing adult survival (after 1st year). Dashed lines indicate the immigration process with ω representing the immigration rate plus noise parameter.

Count data were analyzed using a state‐space matrix population model parameterized by productivity (f) and first‐year ($\alpha_{1}$) and adult ($\alpha_{2}$) apparent survival probabilities to propagate individuals through the system (Figure 2). This model used state process equations to define population size in each year as a function of demographic rates and population size in the preceding year. Process equations including demographic stochasticity were formulated using five classes: first year ($N_{1}$), second year ($N_{2}$), third year ($N_{3}$), and fourth year and older individuals ($N_{4}$) and immigrants ($N_{\mathrm{imm}}$). Assuming a pre‐breeding census, state process equations were defined as:

(1) $$N_{1,t+1}\sim \text{Binomial}\left(\frac{f_{t}}{2}*\alpha_{1,t},N_{\mathrm{tot},t}\right)$$

(2) $$N_{2,t+1}\sim \text{Binomial}\left(\alpha_{2,t},N_{1,t}\right)$$

(3) $$N_{3,t+1}\sim \text{Binomial}\left(\alpha_{2,t},N_{2,t}\right)$$

(4) $$N_{4,t+1}\sim \text{Binomial}\left(\alpha_{2,t},N_{3,t}+N_{\mathrm{tot},t}\right)$$

(5) $$N_{\mathrm{imm},t+1}\sim \text{Poisson}\left(\;\left(N_{1,t}+N_{2,t}+N_{3,t}+N_{4,t}\right)*\omega \right)$$

where omega (*ω*) represents the immigration rate plus noise parameter modeled as a constant proportion of all age classes. We assume only individuals aged 4 and older breed as this is the mean age at which individuals return to the colony based on the CMR data and results of a previous study on MSI (Lavers, Jones, and Diamond 2008; Breton and Diamond 2014). The total breeding population ($N_{\mathrm{tot},t}$) was defined as the total number of breeding‐age individuals ($N_{4,t}$) plus the number of breeding‐age immigrants ($N_{\mathrm{imm},t}$):

(6) $$N_{\mathrm{tot},t}=N_{4,t}+N_{\mathrm{imm},t}.$$

The observation equation then links population size with breeding pair counts (*y*) by accounting for observation error. Counts were modeled using a Poisson distribution defined by $N_{\mathrm{tot},t}$. We modeled only the breeding portion of the population as our count data represent breeding pairs only:

(7) $$y_{t}\sim \text{Poisson}\left(N_{\mathrm{tot},t}\right).$$

Initial population sizes for each age‐class were based on a stable age distribution, initial estimates of population size (approximately 8000 pairs in 2000 for ATPU, 400 pairs in 1998 for RAZO), and estimates of demographic rates in intervening years for ATPU (Whidden 2016; Lavers, Jones, and Diamond 2007; Appendix 1: Figure A2A,B). To test the sensitivity of the model to the assumption of a stable age distribution, we varied the initial population sizes within the range of observed values and found that results were robust to these changes. The initial number of immigrants was defined by a Poisson distribution with a lambda (the mean number of immigrants per year) of 50 for both ATPU and RAZO which represent 0.5% and 10% of the populations, respectively. Here, we assume there is a higher proportion of RAZO immigrants given that the RAZO population on MSI is growing while the ATPU population appears stable. Annual realized population growth rate ($\lambda_{t}$) was calculated as the proportion of $N_{\mathrm{tot},t+1}$ to $N_{\mathrm{tot},t}$.

#### 2.2.2 CMR Data Submodel

A zero‐inflated Gamma‐Poisson model adapted from Riecke et al. (2022) was used to analyze the CMR data. This model accounts for individual heterogeneity in the expected number of observations per individual by modeling the number of times each individual was seen at each time step ($c_{i,t}$, where time step = breeding season), given the mean expected number of encounters per individual (*ε*), availability for detection ($k_{i,t}$), and individual heterogeneity in detection ($h_{i}$), the last being estimated using an overdispersion parameter (*θ*). Thus, we accounted for variation in individual encounter probabilities by summarizing the CMR data as the number of times each individual was encountered during each breeding season, rather than the typical binomial measure of “1” (encountered) and “0” (not encountered). Age was included as a categorical covariate with “1” for first‐year birds, “2” for second‐year birds, “3” for third‐year birds, and “4” for birds that were 4 years or older. However, age was constrained differently depending on the model parameter. We defined first‐year apparent survival probability as survival during the first year of life, and apparent survival of adults as survival probability after the first year of life. This is because first‐year survival is often lower than survival in subsequent years, while survival after the first year is typically closer to adult survival (Lack 1966; Beauchamp 2023). Additionally, given that most young birds do not return until around age 4, there is not enough CMR data to get meaningful annual survival estimates for multiple years prior to age 4. The probability of availability for detection from the research blinds (*γ*) at time *t* given either absence ($\gamma_{1}$) or presence ($\gamma_{2}$) at the previous time step was allowed to vary by age class (first, second, third, and fourth+ year). This allowed us to account for temporary emigration and the gradual return of birds to the colony as they approached breeding age. Finally, the mean expected number of encounters per individual (*ε*) was also modeled as a function of age. Here, pre‐breeding individuals were defined as ages 1–3 while breeding individuals were defined as age 4+ as we expect the mean number of encounters per individual to be similar for pre‐breeding individuals (ages 1–3) and different from breeding individuals (ages 4+).

The latent state of each individual at each time step ($z_{i,t}$) was modeled as a Bernoulli random variable described by the product of apparent survival probability at the previous time step ($\varphi_{i,t-1}$) and the latent state at the previous time step ($z_{i,t-1}$):

(8) $$z_{i,t}\sim \text{Bernoulli}\left(\varphi_{i,t-1}*z_{i,t-1}\right)$$

Annual apparent survival ($\varphi_{i,t}$) was constrained by age with $\alpha_{1}$ = first year survival and $\alpha_{2}$ = adult survival (after first year):

(9) $$\varphi_{i,t}=\alpha_{{\mathrm{age}}_{i,t},t}.$$

To incorporate environmental stochasticity, we allowed survival rates to vary independently using random temporal effects by assuming that the year‐specific realized values were derived from a normal distribution with a mean of zero and variance of 1 (see Data Availability Statement for model code). We then modeled availability for capture of each individual at each time step ($k_{i,t}$) as a Bernoulli random variable defined by the product of the latent state at the same time step ($z_{i,t}$) and the probability of availability for detection (γ) at time *t* given availability at the previous time step ($k_{i,t-1}$) and age at the previous time step (${\mathrm{age}}_{i,t-1}$):

(10) $$k_{i,t}\sim \text{Bernoulli}\left(z_{i,t}*\gamma_{k_{i,t-1}{\mathrm{age}}_{i,t-1}}\right).$$

The observation process modeled the number of times an individual was detected ($c_{i,t}$) as a Poisson random variable defined by the product of availability at the same time step ($k_{i,t}$), the mean expected number of encounters per individual ($\varepsilon_{{\mathrm{age}}_{i,t},t}$ modeled using fixed group and random temporal effects), and an individual heterogeneity ($h_{i}$) given an overdispersion parameter (*θ*):

(11) $$\begin{array}{c} c_{i,t}\sim \text{Poisson}\left(k_{i,t}*\varepsilon_{{\mathrm{age}}_{i,t},t}*h_{i}\right)\\ h_{i}\sim \text{gamma}\left(\theta ,\theta \right) \end{array}$$

This submodel was used to analyze the CMR data alone outside of the IPM framework to compare survival estimates obtained from the independent and integrated approaches. Estimates for detection probability ($p_{\mathrm{age},t}$) were calculated from epsilon ($\varepsilon_{\mathrm{age},t}$) as:

(12) $$p_{\mathrm{age},t}=1-\exp\left(-\left({ɛ}_{\mathrm{age},t}\right)\right)$$

#### 2.2.3 Productivity Data Submodel

The productivity data were analyzed by modeling the annual number of fledglings ($J_{t}$) as a binomial random variable defined by the number of burrows monitored ($B_{t}$) and productivity ($f_{t}$):

(13) $$J_{t}\sim \text{Binomial}\left(f_{t},B_{t}\right).$$

To incorporate environmental stochasticity, we allowed annual productivity to vary independently using random temporal effects by assuming that the year‐specific realized values were derived from normal distributions with a mean of zero and variance that we estimated (see Data Availability Statement for model code).

### 2.3 Model Fitting and Evaluation

We analyzed our IPM using program JAGS (Plummer 2003) called from R (R Core Team 2023) using the package *jagsUI* (Kellner 2021) to produce posterior distributions (see Data Availability Statement for model code and data access). We compared posterior distributions for annual survival and productivity estimates obtained from the IPM to those obtained from separate analyses of each individual dataset to evaluate model performance. All results are reported as posterior distributions summarized by their mean and 95% credible interval. See the Appendix 1 for specific details regarding model fit and component model assumptions.

### 2.4 Post Hoc Analyses

We conducted post hoc tLTREs to determine the demographic drivers of realized population growth rate ($\lambda_{t}$ or, the ratio of population sizes in successive years; Koons et al. 2016, Koons, Arnold, and Schaub 2017; Schaub and Kéry 2021; see Data Availability Statement for model code) using annual estimates of demographic rates ($\alpha_{1,t}$, $\alpha_{2,t}$, and $f_{t}$) and stage/age structured population sizes ($N_{1,t}$, $N_{2,t}$, $N_{3,t}$, $N_{4,t}$, $N_{\mathrm{imm},t}$). For a full description of the tLTRE methods, see Appendix 1. Finally, we conducted post hoc linear regression analyses on the mean estimates for each demographic rate (first‐year survival, adult survival, and productivity) to determine if there was a significant trend over time.

## 3 Results

### 3.1 Demographic Parameter Estimates

**FIGURE 3:**
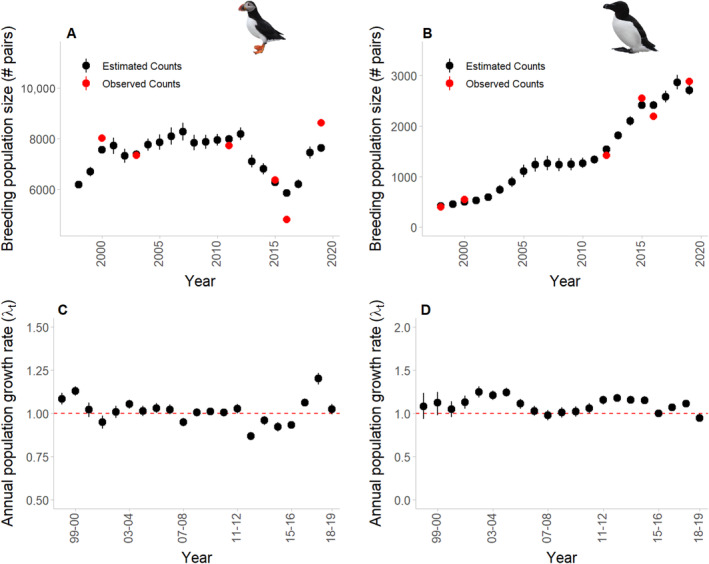
Breeding population size estimates ($N_{\mathrm{tot}}$; in black) from the IPM as compared to the observed population counts (*y*; in red) for (A) ATPU and (B) RAZO. Annual estimates of population growth rate for (C) ATPU and (D) RAZO with red dashed horizontal line indicating a growth rate of 1, or a stable population. Points for estimated counts represent posteriors means with vertical bars representing the 95% credible intervals.

The ATPU breeding population size remained relatively stable at around 8000 pairs (Figure 3A), while the RAZO population increased from approximately 400 to 2700 pairs (Figure 3B); the mean annual growth rate (*λ*) was 1.01 (95% CRI 0.87–1.20, Figure 3C) and 1.10 (95% CRI 0.94–1.26; Figure 3D) for ATPU and RAZO, respectively.

**FIGURE 4:**
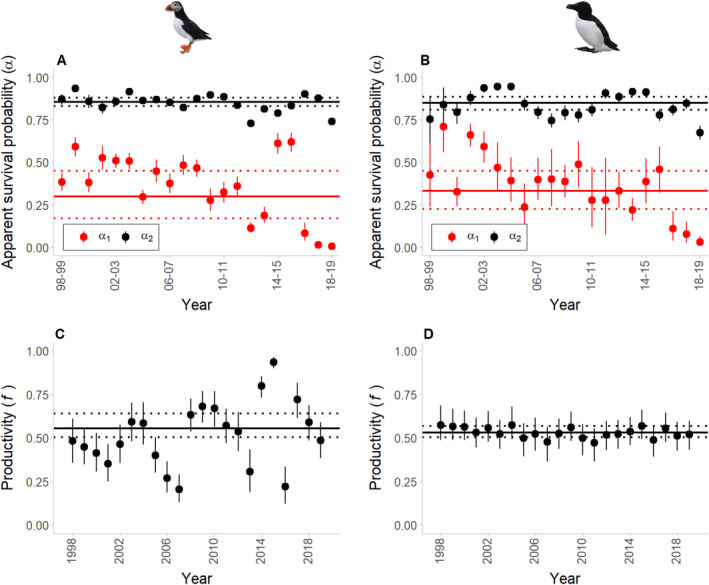
Annual and mean apparent first‐year survival ($\alpha_{1}$; in red), apparent adult (after 1st year) survival ($\alpha_{2}$; in black) for (A) ATPU and (B) RAZO. Annual and mean productivity (*f*) estimates for (C) ATPU and (D) RAZO. Points represent posterior means with 95% credible intervals represented by vertical bars. Solid horizontal lines indicate overall mean and dashed horizontal lines represent 95% credible intervals.

**FIGURE A6:**
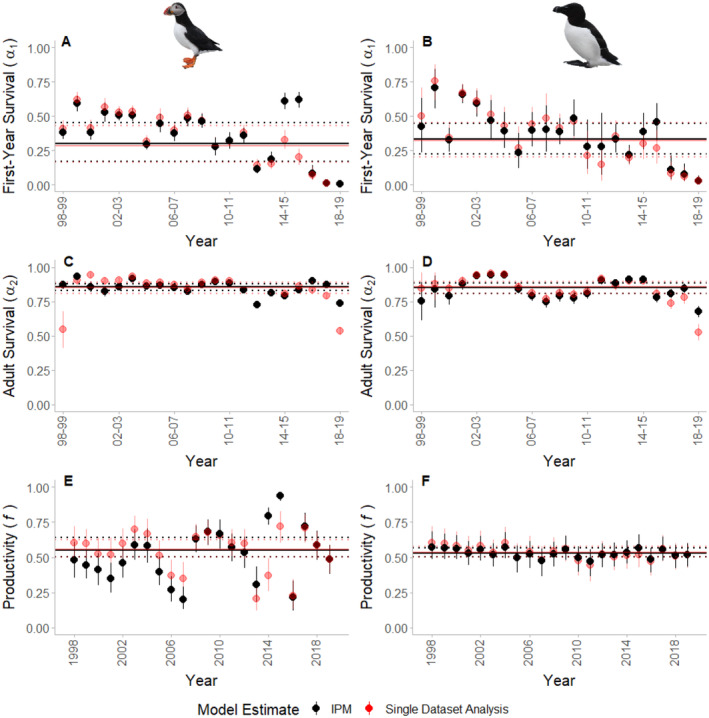
Annual and mean apparent first‐year survival ($\alpha_{1}$), apparent adult (after 1^st^ year) survival ($\alpha_{2}$), and productivity (f) estimates for ATPU and RAZO comparing those obtained from IPM (black) to those from single data set analyses (red). (A) ATPU first‐year apparent survival, (B) RAZO first‐year apparent survival, (C) ATPU apparent adult (after 1^st^ year) survival, (D) RAZO apparent adult (after 1^st^ year) survival, (E) ATPU productivity, and (F) RAZO productivity. Points represent posterior means with 95% credible intervals represented by vertical bars. Solid horizontal lines indicate overall mean and dashed horizontal lines represent 95% credible intervals.

**FIGURE A7:**
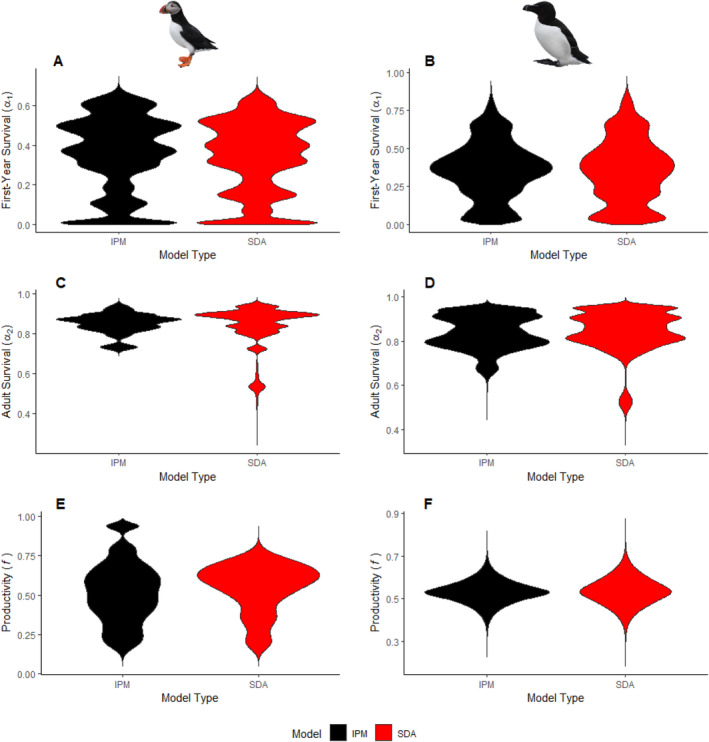
Distribution of annual estimates of apparent first‐year survival ($\alpha_{1}$), apparent adult (after 1^st^ year) survival ($\alpha_{2}$), and productivity (*f*) estimates for ATPU and RAZO comparing those obtained from IPM (black) to those from single data set analyses (red). (A) ATPU first‐year apparent survival, (B) RAZO first‐year apparent survival, (C) ATPU apparent adult (after 1^st^ year) survival, (D) RAZO apparent adult (after 1^st^ year) survival, (E) ATPU productivity, and (F) RAZO productivity.

Excluding the last three estimates due to low return rates of first‐year birds, annual ATPU first‐year survival ($\alpha_{1}$) ranged from 0.11 (95% CRI 0.08–0.15) to 0.62 (95% CRI 0.56–0.67) with a mean of 0.30 (95% CRI 0.17–0.45; Figure 4A). Results from the post hoc linear regression show no significant trend (i.e., stable over the study period; slope: −0.006; *p*‐value: 0.412). Annual estimates of ATPU adult survival ($\alpha_{2}$) ranged from 0.73 (95% CRI 0.71–0.75) to 0.93 (95% CRI 0.91–0.95) with a mean of 0.85 (95% CRI 0.83–0.88; Figure 4A) and an overall weakly significant negative trend over time (slope: −0.004; *p*‐value: 0.046). Excluding the last estimate, annual estimates of RAZO first‐year survival showed more uncertainty than ATPU and ranged from 0.22 (95% CRI 0.16–0.29) to 0.71 (95% CRI 0.56–0.84) with a mean of 0.33 (95% CRI 0.22–0.45; Figure 4B). Results of the post hoc linear regression show a weakly significant negative trend in RAZO first‐year survival over time (slope: −0.013; *p*‐value: 0.032). Annual RAZO adult survival ranged from 0.75 (95% CRI 0.70–0.79) to 0.95 (95% CRI 0.93–0.97) with a mean of 0.85 (95% CRI 0.81–0.89; Figure 4B) and an overall nonsignificant trend over the study period (slope: −0.002; *p*‐value: 0.456). Finally, annual estimates of ATPU productivity (*f*) were highly variable, ranging from 0.20 (95% CRI 0.13–0.29) to 0.94 (95% CRI 0.90–0.96) with a mean of 0.55 (95% CRI 0.50–0.64; Figure 4C). Estimates for RAZO were more consistent, ranging from 0.47 (95% CRI 0.36–0.55) to 0.57 (95% CRI 0.49–0.68) with a mean of 0.53 (95% CRI 0.50–0.57; Figure 4D). Results of the post hoc linear regression reveal no significant trend over the study period for either ATPU (slope: 0.009; *p*‐value: 0.158) or RAZO (slope: −0.002; *p*‐value: 0.095). The range and mean of annual estimates obtained from analyses of the individual datasets (single dataset analyses) generally matched with those obtained from the IPM (Appendix 1: Figures A6 and A7), indicating relative agreement between the datasets. Mean demographic rate estimates were generally at the lower range of values reported in the literature for both species (Appendix 1: Table A2).

**FIGURE A8:**
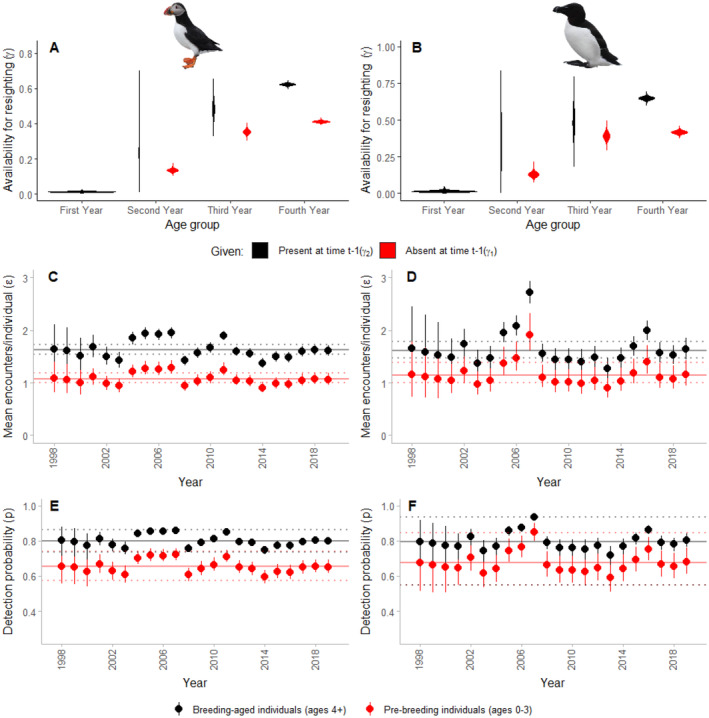
Availability around the research blinds for detection for resighting at time *t* (gamma; *γ*) given absent at time *t*−1 ($\gamma_{1}$; shown in red) and present at time *t*−1 ($\gamma_{2}$; shown in black) for (A) ATPU and (B) RAZO. Mean expected number of encounters per individual (epsilon; *ɛ*) given available to be detected for (C) ATPU and (D) RAZO with breeding‐aged individuals (ages 4+) shown in black and pre‐breeding individuals (ages 0–3) shown in red. Detection probability (*p*) given available to be detected for (E) ATPU and (E) RAZO again with breeding‐aged individuals (ages 4+) shown in black and pre‐breeding individuals (ages 0–3) shown in red. For panels C through F, points represent annual posterior means with 95% credible intervals represented by vertical bars. Solid horizontal lines indicate overall mean and dashed horizontal lines represent 95% credible intervals.

Estimates of probability of availability for detection (γ) for both species were higher if the individual was available for detection at the previous time step (Appendix 1: Figure A8A,B; Table A3). Availability for detection (*γ*) increased with age for both species, and the mean expected number of encounters per individual (*ɛ*; Appendix 1: Figure A8C,D; Table A3) and consequently detection probability (*p*; Appendix 1: Figure A8E,F; Table A3) were also higher in older birds than younger birds for both species.

The overdispersion parameter (*θ*) defining individual heterogeneity ($h_{i}$) was estimated at 39.0 (95% CRI 29.5–52.5) for ATPU and 16.2 (95% CRI 12.1–22.0) for RAZO, indicating that individual heterogeneity in detection was higher for RAZO. The immigration plus noise parameter (*ω*) was estimated at 0.08 (95% CRI 0.07–0.09) for ATPU and 0.21 (95% CRI 0.19–0.23) for RAZO, suggesting that per capita immigration was higher for RAZO.

### 3.2 tLTRE

**FIGURE 5:**
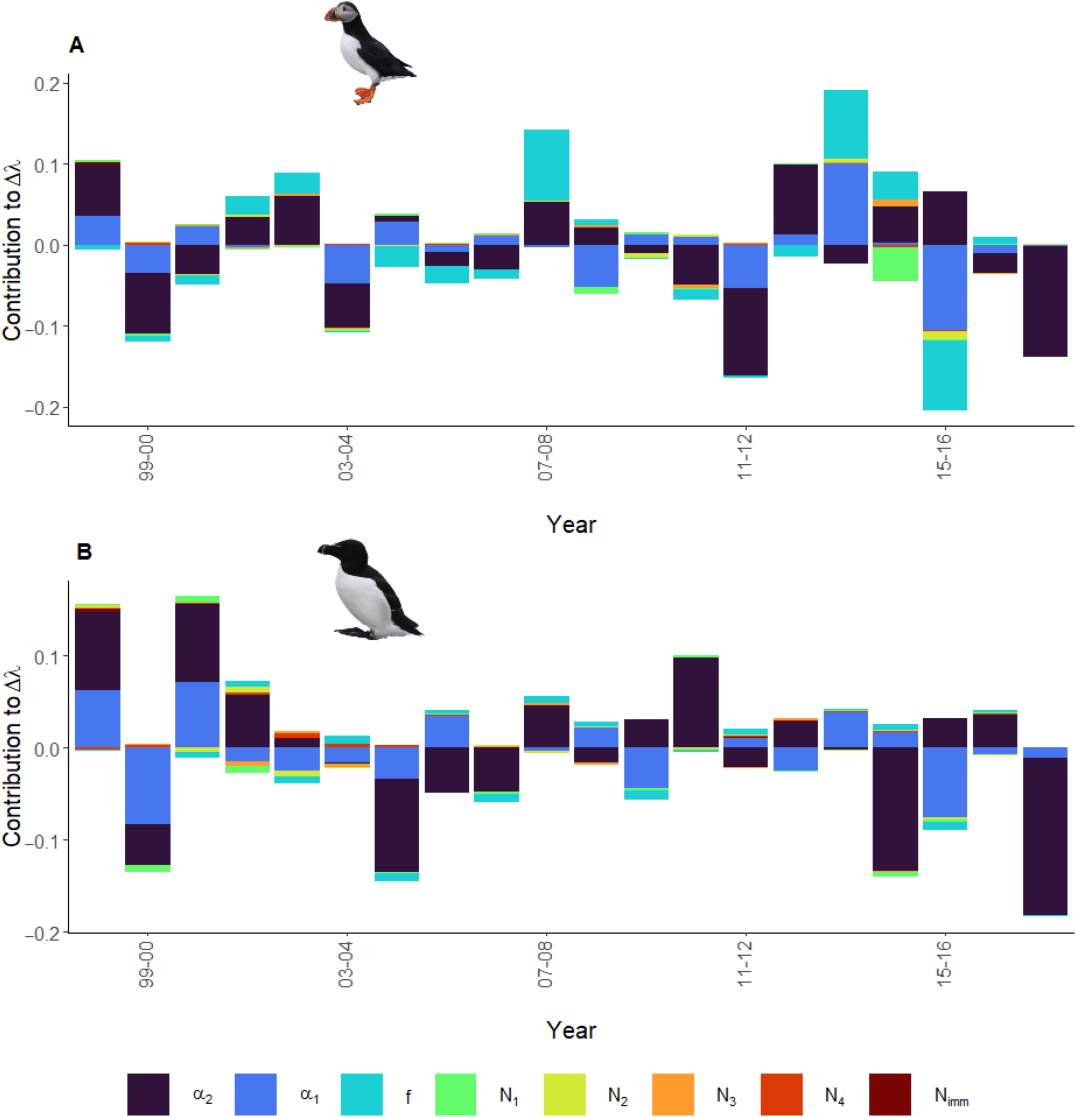
Contributions of annual changes in demographic parameters to annual changes in population growth rate for (A) ATPU and (B) RAZO. $\alpha_{2}$ = adult (after 1st year) survival (dark blue), $\alpha_{1}$ = first‐year survival (blue), f = productivity (teal), $N_{1}$ = # of 1st year individuals (green), $N_{2}$ = # of 2nd year individuals (yellow), $N_{3}$ = # of 3rd year individuals (orange), $N_{4}$ = #of 4th + year individuals (red), and $N_{\mathrm{imm}}$ = # of immigrants (dark red). The years on the *x*‐axis represent the successive years being compared. Negative values indicate the contribution (of that demographic parameter worked) to decrease population growth over the successive time steps.

**FIGURE A9:**
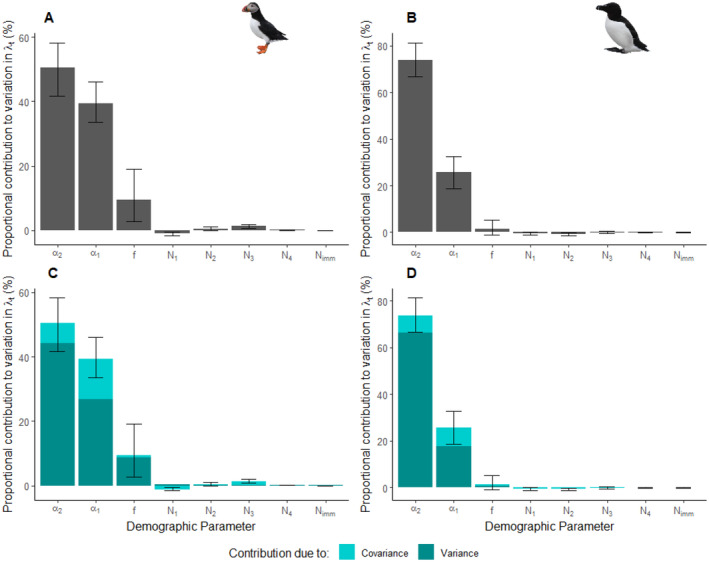
Results of tLTRE showing mean proportional contribution (%) of demographic parameters to variance in population growth for (A) ATPU and (B) RAZO and same mean proportional contribution broken down into contributions due to variance (dark teal) in demographic rate and covariance (light teal) with other demographic parameters for (C) ATPU and (D) RAZO where $\alpha_{2}$ = adult (after 1^st^ year) survival, $\alpha_{1}$ = first‐year survival, f = productivity, $N_{1}$ = # of 1^st^ year individuals, $N_{2}$ = # of 2^nd^ year individuals, $N_{3}$ = # of 3^rd^ year individuals, $N_{4}$ = #of 4^th^ + year individuals, and $N_{\mathrm{imm}}$ = # of immigrants. Error bars represent 95% credible intervals.

**FIGURE A10:**
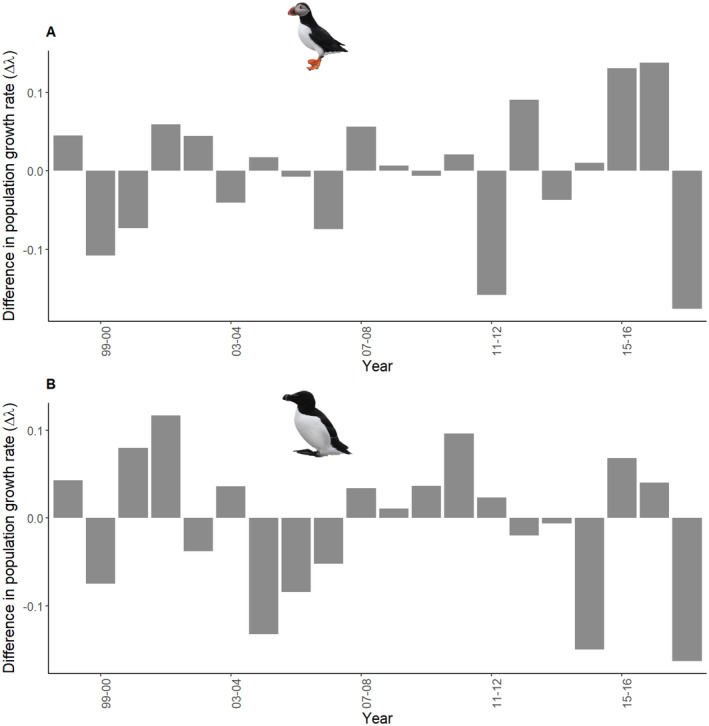
Results of tLTRE showing successive changes in annual population growth for (A) ATPU and (B) RAZO. The years on the x‐axis represent the two successive years being compared. Negative values indicate that annual population growth rate decreased between successive years.

Variability in adult survival contributed the most to variance in population growth rate for both species at approximately 51% for ATPU and 74% for RAZO. This was mostly due to variance in adult survival rather than covariance with other demographic parameters (Appendix 1: Figure A9; Table A4). Variability in first‐year survival and productivity contributed second‐ and third‐most to variance in population growth rate at approximately 39% and 10% for ATPU and 26% and 2% for RAZO, respectively. Again, most of the contribution was due to variance in the demographic parameter rather than covariance with other parameters (Appendix 1: Figure A9; Table A4). Note that negative contribution indicates a reduction in variance in population growth rate rather than contribution. The process correlation, which provides insight into how much demographic rates fluctuate over time in tandem or independently from each other, between adult survival and productivity was −0.06 (−0.20 to 0.08) for ATPU and 0.04 (−0.33 to 0.39) for RAZO. Between adult and first‐year survival, process correlations were 0.27 (0.19–0.35) for ATPU and 0.24 (0.03–0.45) for RAZO. Finally, process correlations between the first‐year survival and productivity were 0.12 (−0.02 to 0.25) for ATPU and 0.23 (−0.18 to 0.57) for RAZO. Successive annual changes in adult survival contributed most to successive annual changes in population growth rate for both species, while first‐year survival contributed the second most (Figure 5; Appendix 1: Figure A10). For ATPU, changes in adult survival contributed the most in 65% of years (or 13 years out of 20) while changes in first‐year survival contributed the most in 25% of years (or 5 years out of 20; Figure 5A). For RAZO, changes in adult survival contributed the most in 65% of years (or 13 years out of 20) while changes in first‐year survival contributed the most in 35% of years (or 7 years out of 20; Figure 5B).

## 4 Discussion

Comparing population dynamics of sympatric species residing at the southern edges of their breeding distributions provides important insights into how they may be responding to shared and rapidly changing environmental conditions. Previous population analyses have been conducted for ATPU and RAZO in the Gulf of Maine (GoM; Breton, Diamond, and Kress 2005; Breton, Diamond, and Kress 2006a; Lavers et al. 2008), but those studies used shorter time‐series that did not encompass the prey regime shift in 2010 and more recent marine heatwave events in 2013 and 2016 (Mills et al. 2013; Pershing et al. 2018). Here, we estimated and compared long‐term (1998–2019) population dynamics for these two species, and explicitly estimated first‐year survival of ATPU and RAZO on Machias Seal Island (MSI) in the GoM. While estimates of first‐year survival were relatively imprecise compared to adult survival, these represent the first estimates of this critical life stage for both species in the northwestern Atlantic region. Overall, we found divergent long‐term population trends between the two sympatric species despite similar mean demographic rates.

Estimates of mean adult survival for ATPU and RAZO from our study were lower than estimates from previous studies. For the MSI populations, Breton and Diamond (2014) estimated mean adult survival of ATPU at 0.92 (SE 0.01) while estimated mean adult survival of RAZO at 0.91 (SE 0.03). Estimates of adult survival in either species from other colonies in the GoM are limited; however, Breton, Diamond, and Kress (2005) estimated mean adult survival for ATPU nesting on Eastern Egg Rock at 0.95 (SE 0.01). In comparison to our analyses, these previous studies covered shorter timeframes that did not include more recent years corresponding to decreased adult survival in our study (i.e., post 2011) and the aforementioned ecosystem changes in the GoM. Studies of the eastern Atlantic populations of these species found mean adult survival to be approximately 0.90 for both ATPU and RAZO (Sandvik et al. 2005; St. John Glew et al. 2019; Newman et al. 2021). This may suggest that the southern‐edge MSI populations are experiencing negative impacts due to rapid warming in the GoM. However, other threats, such as over‐fishing and bycatch, cannot be ruled out and require further investigation.

To date, no long‐term studies have explicitly estimated first‐year survival in ATPU or RAZO in the GoM. Earlier studies of the MSI populations estimated survival from age 0 to 3 at 0.70 (SE 0.02) for ATPU (Breton, Diamond, and Kress 2006a) and survival from age 0 to 2 at 0.78 (SE 0.04) for RAZO (Lavers et al. 2008). Unsurprisingly, we found that mean first‐year survival (ages 0–1) was lower than these previous estimates for all pre‐recruitment years combined. Mortality rates are generally higher in the first year of life, likely due to inexperience in finding food (Lack 1954; Wunderle 1991), while post‐first‐year survival is typically closer to that of adults in long‐lived organisms (Lack 1966; Beauchamp 2023). For long‐lived seabird species with low productivity, first‐year survival is typically estimated around 0.50 while subsequent survival is estimated around 0.80–0.90. This has been found in several seabird species such as Cory's shearwaters (*Calonectris Diomedea*; Jenouvrier et al. 2008), Northern Gannets (*Morus bassanus*; Warwick‐Evans, Green, and Atkinson 2016), Herring Gulls (*Larus argentatus*; Kentie et al. 2023), and the closely related Common Murre (*Uria aalge*; Sarzo et al. 2019). However, because immature ATPU and RAZO do not recruit until around age 4 (and therefore observers cannot resight them in order to get survival estimates), estimates of first‐year survival in the last 3 years of this study are almost certainly underestimated and could be thought of as the bare minimum based on the few individuals that were resighted at ages 1, 2, and 3 (i.e., survival rate is at least this high and likely no lower). As such, mean first‐year survival is more likely toward the upper end of the 95% CRI, in line with what has been reported in the literature for first‐year survival in seabirds. As with the GoM, estimates of first‐year survival for these species in other parts of their range are limited, especially from long‐term studies. However, first‐year survival has been estimated in shorter‐term studies from the Eastern Atlantic. For example, Sandvik et al. (2008) estimated first‐year survival in two ATPU cohorts on Hornøya (Northern Norway) and found very high survival rates, similar to adults at approximately 0.90. However, this study only examined survival rates of two cohorts and had substantial marker loss. As such, the differences seen between our study and Sandvik et al. (2008) may be due to site effects, different rates of permanent emigration, or a number of other factors. In contrast, Steventon (1982) estimated first‐year survival in RAZO on the Shiant Islands (Outer Hebrides) and found it to be no less than 0.16. However, this study used plastic color bands which tend to fade or fall off more easily than metal bands (Breton, Diamond, and Kress 2006b). Additionally, this study did not account for effort or resighting/trapping efficiency. As such, estimates of first‐year survival in that study may be underestimated. More studies are needed for these species across their range to understand long‐term trends in first‐year survival.

Productivity estimates for both species in this study generally aligned with previous estimates from MSI and other colonies in the GoM. Major et al. (2021) estimated mean productivity on MSI between 1995 and 2019 at 0.55 ± 0.06 for ATPU and 0.54 ± 0.04 for RAZO. In comparison, average annual productivity from the smaller colony of Matinicus Rock in the GoM was estimated at 0.65 ± 0.20 (Kress, Shannon, and O'Neal 2016) for ATPU and 0.48 ± 0.13 for RAZO (Kauffman 2014). Similarly, an estimate of average annual productivity for ATPU nesting on Seal Island National Wildlife Refuge (SINWR) in the GoM was found to be approximately 0.66 ± 0.27 (Kress, Shannon, and O'Neal 2016). The findings from these studies also suggest that, similar to our results, annual productivity in ATPU is more variable than RAZO. Estimates of productivity for these species from the Eastern Atlantic have been found to be slightly higher at approximately 0.67–0.70 (Lahoz‐Monfort et al. 2017; Landsem et al. 2023), suggesting that overall productivity is lower in the GoM as compared to other parts of their range. Similar to findings from the Northwestern Atlantic, ATPU productivity in the Eastern Atlantic has been found to be more variable from year to year than RAZO (Lahoz‐Monfort et al. 2013, 2017). These results may be explained by the fact that RAZO productivity has been found to be less sensitive to changes in prey availability than ATPU productivity (Furness and Tasker 2000) due to their ability to locate and deliver high‐quality prey to their chicks while avoiding low‐quality prey (Scopel et al. 2019). Tracking and diet studies reveal that RAZO typically forage in shallow, coastal waters on higher quality prey as compared to ATPU (Pratte, Robertson, and Mallory 2017; Symons and Diamond 2022). This foraging behavior in RAZO may lead to less temporal variability in chick diet (Scopel et al. 2019) and perhaps, less temporal variability in productivity.

Consistent with field observations and count data, our IPM suggests the ATPU population on MSI is relatively stable, while the RAZO population is increasing. The trends in breeding population size observed in this study agree with the long‐term trends across eastern Canada for both species, suggesting that the population trends on MSI are largely consistent with species‐level trends in the region. The average trend from 1970 to 2016 for ATPU in Eastern Canada was a 1.2% (−0.3% to 2.7%) increase per year and a 4.8% (2.7%–6.7%) increase per year for RAZO (Environment and Climate Change Canada 2019). Interestingly, global population trends suggest that ATPU are in decline across the eastern Atlantic and are listed as “vulnerable” at the global level (BirdLife International 2018) and “endangered” in Europe (BirdLife International 2021c). However, RAZO populations appear to be increasing leading to a listing of “least concern” both globally (BirdLife International 2021a) and in Europe (BirdLife International 2021b). Our findings for ATPU from the current study are especially concerning given that the global population is in decline. If we continue to see decreases in ATPU survival on MSI, then we may expect to see a similar population decline in the future.

Results of the tLTREs indicate that variance/covariance in adult survival contributed most to variance in population growth rate. Most of this contribution was due to variance in adult survival rather than covariance with other demographic parameters. Similarly, annual changes in adult survival contributed the most to annual changes in population growth rate. Our findings agree with predictions of growth rate sensitivity for long‐lived species with delayed maturity and small clutch sizes (Lebreton and Clobert 1991; Pfister 1998; Sæther and Bakke 2000; Stahl and Oli 2006), including seabird species such as Marbled Murrelets (*Brachyramphus marmoratus*; Boulanger et al. 1999), Snow Petrels (*Pagodroma nivea*), Emperor Penguins (*Aptenodytes forsteri*; Jenouvrier, Barbraud, and Weimerskirch 2005), and albatross (family Diomedeidae; Pardo et al. 2017). These results are consistent with life‐history theory, where breeding individuals should attempt to optimize their lifetime reproductive output by maximizing their own survival and minimizing reproductive costs (Drent and Daan 1980; Partridge 1989; Ricklefs 1990; Stearns 1992; Erikstad et al. 1998). As such, even slight decreases in adult survival can have large impacts on the lifetime reproductive success of individuals (Wooller, Bradley, and Croxall 1992) and therefore population growth rate.

Overall, the temporal trends observed in the demographic rates indicate the possibility of a long‐term decline in ATPU adult survival and RAZO first‐year survival. However, further studies are needed to understand the magnitude and drivers of these trends. As such, continued monitoring of these populations is warranted to track changes and trends in demographic rates. Given that adult survival was the primary driver of population dynamics in these species, and that small changes in adult survival can result in proportionately large changes in population growth rate, future monitoring and conservation efforts should be focused outside the breeding season in critical overwintering areas and migratory locations in the GoM (Baran et al. 2022; Dodds 2024) where adult mortality is typically concentrated (Sandvik et al. 2005; Clairbaux et al. 2021). This is especially important for ATPU given that our results indicate a possible long‐term decline in this demographic rate. While first‐year survival was the secondary driver of population dynamics in these species, survival of young birds is necessary for recruitment into the population and therefore overall population dynamics (Sæther et al. 2013). Additionally, conditions experienced during the early part of life can potentially have long‐term fitness consequences for individuals (Lindström 1999; Metcalfe and Monaghan 2001; Cam and Aubry 2011; Fay et al. 2015). As such, additional monitoring and tracking efforts are warranted to understand where these immature individuals are going after they fledge from the colony. This is especially important for RAZO given that our results indicate a possible long‐term decline in first‐year survival. Similarly, high interannual variability in productivity can influence recruitment into the population, which may eventually impact overall population dynamics (Jenouvrier et al. 2005; Sandvik, Erikstad, and Sæther 2012). Therefore, future monitoring and conservation efforts should be focused on identifying drivers of productivity and strategies to increase overall breeding success in these populations.

In summary, we found that the ATPU population on MSI remained relatively stable over the 22‐year study period, whereas the RAZO population increased substantially. Estimates of mean survival and productivity were similar between the study species but were at the lower range of values reported in the literature across their range. These findings suggest that the MSI population may be experiencing negative impacts from the rapid warming in the GoM, but future studies are needed to identify specific drivers of demographic changes. Despite similar estimates of mean productivity, interannual variation in this demographic rate was much higher in ATPU than RAZO. These results may be explained by differential responses to shared environmental conditions as a result of differences in life history strategy and niche separation. Adult survival was found to be the primary driver of population growth for both species but shows evidence of a long‐term decline in ATPU. While the observed decline in demographic rates might be due to ecosystem changes or other anthropogenic factors, these species appear to be responding somewhat asynchronously. Importantly, extreme climate events such as marine heatwaves often produce conditions like those predicted to continue as the ocean warms (Brickman et al. 2021; Woehler and Hobday 2024). Thus, the demographic responses observed during these events may be analogous to future responses as ocean temperatures continue to rise (Pershing et al. 2021). Taken together, these results are concerning, as continued decreases in survival over time would inevitably result in decreased population sizes and a higher risk of extinction. Thus, while we are not currently seeing population declines given that the ATPU population is stable and the RAZO population is increasing, we could expect populations to begin to decline in the coming years if demographic rates continue to decline. Future work examining population viability under various climate scenarios will continue to inform our understanding of how these southern range‐edge populations are responding to a rapidly warming planet.

## Author Contributions

**Sarah E. Durham:** conceptualization (lead), data curation (supporting), formal analysis (lead), investigation (lead), methodology (lead), project administration (lead), writing – original draft (lead), writing – review and editing (equal). **Sarah P. Saunders:** formal analysis (supporting), investigation (supporting), methodology (supporting), writing – review and editing (equal). **Antony W. Diamond:** conceptualization (supporting), data curation (lead), formal analysis (supporting), investigation (supporting), methodology (supporting), writing – review and editing (equal). **Thomas V. Riecke:** formal analysis (supporting), investigation (supporting), methodology (supporting), writing – review and editing (equal). **Heather L. Major:** conceptualization (supporting), data curation (lead), formal analysis (supporting), funding acquisition (lead), investigation (supporting), methodology (supporting), project administration (supporting), writing – review and editing (equal).

## Conflicts of Interest

The authors declare no conflicts of interest.

## Statement of Inclusion

Our study includes authors from both countries in which the study area is located. No data were collected specifically for this project, however, all authors were consulted early in the data analysis phase to ensure inclusion of different perspectives and expertise.

## Figures and Tables

**FIGURE A5:**
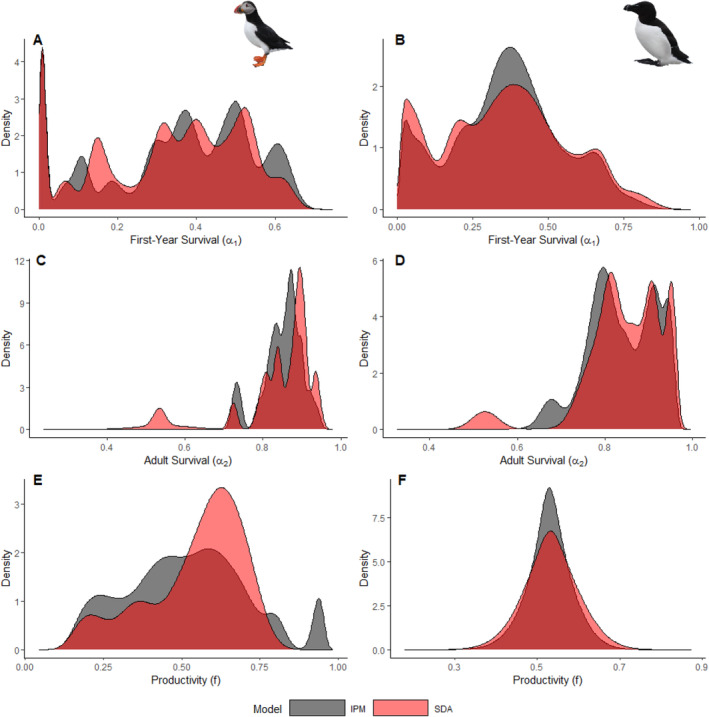
IPM (gray) and single data set analyses (red) posterior distribution overlap for (A) ATPU first‐year survival ($\alpha_{1}$; 85.5%), (B) RAZO first‐year survival ($\alpha_{1}$; 91.7%), (C) ATPU adult (after 1st year) survival ($\alpha_{2}$; 70.4%), (D) RAZO adult (after 1st year) survival ($\alpha_{2}$; 85%), (E) ATPU productivity (*f*; 77%), and (F) RAZO productivity (*f*; 88.9%).

## Data Availability

The model code to run these analyses can be obtained from the GitHub Repository at https://github.com/SEDurham/ATPU_RAZO_IPM. A temporary link to the data that support the findings of this study is available from the UNB Dataverse repository at https://dataverse.lib.unb.ca/privateurl.xhtml?token=b5f3152f‐a5fa‐4f53‐a631‐b27dcadce6de. A DOI has been assigned to this dataset and will remain unchanged: Durham, Sarah; Diamond, Antony; Major, Heather, 2024, “Datasets for Atlantic Puffin and Razorbill Integrated Population Models”, https://doi.org/10.25545/8W7JSF, UNB, DRAFT VERSION. The above DOI will be published and data made available upon request from the corresponding authors, Dr. Heather Major (heather.major@unb.ca) or Dr. Tony Diamond (diamond@unb.ca) once the review is completed and all corrections are made to the dataset.

## Appendix 1

### Model Fitting and CMR Goodness of Fit

We specified vague priors for most parameters of interest except for the immigration rate plus noise parameter (omega; *ω*) as this parameter had very little data to inform the estimate. As such, we restricted the prior to a beta distribution defined by *α* = 3 and *β* = 7 given we know there is some, but very limited, immigration of both species into the MSI population (Lavers, Jones, and Diamond 2007; Whidden 2016). For the ATPU model, we ran three chains with a thinning rate of 5, with 1,000,000 iterations, a burn‐in phase of 250,000, and an adaptive phase of 50,000; this resulted in 450,000 values sampled for each model parameter. For the RAZO model, we ran three chains with a thinning rate of 5, with 500,000 iterations, a burn‐in phase of 150,000 and an adaptive phase of 25,000 resulting in 210,000 values sampled for each model parameter. Model convergence was determined using the R‐hat statistic (Gelman and Hill 2007; Gelman et al. 2014) and visual inspection of chains. Overall goodness of fit was determined by calculating the percent overlap in the posterior distributions of the IPM estimates for survival and productivity as compared to the analyses of the single data sets. We assumed that an overlap of > 75% for parameters of interest indicated that the models were not misspecified (Plard, Turek, and Schaub 2021). The posterior distribution overlap percentage between the IPM estimates and the single data set analysis estimates for the parameters of interest were mostly above the 75% threshold (Figure A5). Posterior distribution overlap percentage for adult survival was slightly below this threshold but is likely due to minimal resights in 1999 leading to low estimates from the CMR only analysis.

Goodness of fit tests were performed on the CMR data prior to model construction using the *R2ucare* package (Gimenez et al. 2018). The results of the goodness of fit tests (Table A1) suggested that a capture‐mark‐recapture model should be fit that incorporates trap‐dependence, transience, and overdispersion. A significant and negative test statistic for test 2.CT suggests that there is an excess of individuals encountered at a given occasion among the individuals encountered at the previous occasion. A significant and positive test statistic for test 3.SR suggests that newly marked individuals have a lower probability of being resighted in the future than previously marked individuals (Gimenez et al. 2018).

Transience effects in CMR data may result from different biological causes or may be due to the marking procedure. For some species like seabirds, survival and permanent dispersal may be different for younger individuals which can show up in the data as a transient effect (Genovart and Pradel 2019). As such, incorporating age structure into the models through the use of separate juvenile and adult survival rates addresses this violation of assumption of the classic CJS model (Pradel et al. 1997). Trap dependence in the data is most likely an artifact of study design and methods for resighting individuals. Birds are resighted each season from established research blinds that are placed in specific, dense locations within the colony. As such, birds nesting close to the blinds tend to be resighted more than birds nesting further away from the blinds which violates the assumption of equal catchability. This is a form of heterogeneity in detection probability and causes variation in the latent encounter probabilities of individuals (Riecke et al. 2022) due to where the sampling effort is focused (Royle and Young 2008). This form of trap‐dependence has been found in other study systems of the same species (Lahoz‐Monfort et al. 2011). Overdispersion is caused by a lack of independence in the observation process and likely results from the fact that some individuals are seen many times over the course of the breeding season while others are seen rarely if ever (also stems from focus of sampling effort). The result of overdispersion is that error surrounding survival estimates will be much too small (Pradel, Gimenez, and Lebreton 2005; Afroz, Parry, and Fletcher 2019) but does not bias survival estimates (Schmidt, Schaub, and Anholt 2002). Both overdispersion and trap‐dependence as indicated by the GOF tests can be addressed by accounting for heterogeneity in the observation process (Riecke et al. 2022).

**TABLE A1:** Results of goodness of fit tests performed within the R2ucare package assessing the fit of the CMR data to the CJS model. The overall CJS test assess the overall fit of the CJS model. Test 2. CT tests for the presence of transience while test 3. SR tests for the presence of trap dependence. Test 3. SM assess whether, among individuals seen again, if when they were seen differs among previously and newly marked individuals. Test 2. CL assess whether there is a difference in the timing of reencounters between the individuals encountered and not encountered at occasion *t*, conditional on the presence at both occasions *t* and *t* + 2.

Species	Test	*χ* ^2^	df	*p*	Test sign
ATPU	Overall CJS	3505.469	277	0	NA
	2.CT	680.218	19	0	−20.779
	3.SR	1252.113	20	0	30.713
	3.SM	1198.886	104	0	NA
	2.CL	374.272	134	0	NA
RAZO	Overall CJS	1213.007	182	0	NA
	2.CT	220.679	19	0	−10.732
	3.SR	350.981	20	0	16.027
	3.SM	510.69	54	0	NA
	2.CL	130.657	86	0.001	NA

### Post Hoc Analyses: Transient Life Table Response Experiments (tLTREs)

Temporal variance of $\lambda_{t}$ was decomposed into components due to the variance/covariance of the demographic parameters (i.e., demographic rates and population structure) by calculating the sensitivity of $\lambda_{t}$ to changes in the demographic parameters (evaluated at the mean of the demographic parameters for each MCMC draw). The variance–covariance matrix was calculated using annual estimates of the demographic parameters and the sensitivities and covariances were used to estimate the contribution of the variability in each demographic parameter to the temporal variance of $\lambda_{t}$

(A1)
$${\text{Contribution}}_{\Theta_{i}}^{\mathrm{var}\left(\lambda_{t}\right)}\approx \sum_{j}\mathrm{cov}\left(\Theta_{i,t},\Theta_{j,t}\right)\frac{\partial \lambda_{t}}{\partial \Theta_{i,t}}\frac{\partial \lambda_{t}}{\partial \Theta_{j,t}}|\theta \;\theta \;\underline{\Theta_{i,t}}$$

where $\Theta_{t}$ is a vector of time specific demographic rates and proportional stage/age structured population sizes. Proportional contributions to the variance of $\lambda_{t}$ were calculated as the contribution of each demographic parameter to the total contribution. We then used the temporal variance of each demographic rate ($\sigma^{2}$) and the temporal covariance between them (σ) from the variance–covariance matrix (Σ):

(A2)
$$\sum =\left[\begin{array}{ccc} \sigma_{\alpha_{1}}^{2} & \sigma_{\alpha_{1},\alpha_{2}} & \sigma_{\alpha_{1},f}\\ \sigma_{\alpha_{2},\alpha_{1}} & \sigma_{\alpha_{2}}^{2} & \sigma_{\alpha_{2},f}\\ \sigma_{f,\alpha_{1}} & \sigma_{f,\alpha_{2}} & \sigma_{f}^{2} \end{array}\right]$$

to calculate the process correlations (*ψ*) between demographic rates:

(A3)
$$\psi_{\alpha_{1},\alpha_{2}=\frac{\sigma_{\alpha_{1},\alpha_{2}}}{\sqrt{\sigma_{\alpha_{1}}^{2}\sigma_{\alpha_{2}}^{2}}}}\;\psi_{\alpha_{1},f=\frac{\sigma_{\alpha_{1},f}}{\sqrt{\sigma_{\alpha_{1}}^{2}\sigma_{f}^{2}}}}\;\psi_{\alpha_{2},f=\frac{\sigma_{\alpha_{2},f}}{\sqrt{\sigma_{\alpha_{2}}^{2}\sigma_{f}^{2}}}.}$$

Process correlations provide insight into how much demographic rates fluctuate over time in tandem or independently from each other. To determine the contribution of successive annual differences in the demographic parameters to successive annual differences in $\lambda_{t}$, we calculated the differences and means of the demographic parameters between successive time steps. We then calculated the sensitivities, which were evaluated at the mean values between successive years rather than the means for each MCMC draw. The contribution was calculated as the product of the differences and sensitivities:

(A4)
$${\text{contribution}}_{\Theta_{i}}^{\Delta \lambda_{t}}\approx \left(\Theta_{i,t+1}-\Theta_{i,t}\right)\frac{\partial \lambda_{t}}{\partial \Theta_{i,t}}|\theta \;\theta \;\underline{\Theta_{i}}$$

### Demographic Rate Comparisons Between This Study and Published Estimates

**TABLE A2:** Mean and variability of survival and productivity for Atlantic Puffins and Razorbills at Machias Seal Island and other colonies across their breeding ranges. All estimates reported as mean value unless otherwise noted.

Species/Location	Years	Demographic rate	Estimate	Source
ATPU				
East coast of UK^a^	2014–2018	Productivity	0.727 (95% CI: 0.623–0.793)^b^	Searle et al. (2022)
Fair Isle (Shetland)	1987–2013	Productivity	0.62 (SD: 0.18)^c^	Miles et al. (2015)
Hernyken (Røst, Norway)	1990–2020	Productivity	0.086 (0.0–0.959)^d^	Landsem et al. (2023)
Hornøya (Finnmark, Norway)	1990–2020	Productivity	0.772 (0.129–0.925)^d^	Landsem et al. (2023)
Isle of May (Scotland)	1990–2020	Productivity	0.67 (0.30–0.84)^d^	Landsem et al. (2023)
Machias Seal Island (Gulf of Maine)	1998–2019	Productivity	0.55 (0.50–0.64)	This study
Machias Seal Island (Gulf of Maine)	1995–2019	Productivity	0.55 (0.49–0.61)	Major et al. (2021)
Matinicus Rock (Gulf of Maine)	2004–2014	Productivity	0.65 (0.45–0.85)	Kress, Shannon, and O'Neal (2016)
Seal Island (Gulf of Maine)	2006–2014	Productivity	0.66 (0.39–0.93)	Kress, Shannon, and O'Neal (2016)
Hornøya (Finnmark, Norway)	1994–1995	Survival age 0–1	0.920 (95% CI: 0.742–0.979)	Sandvik et al. (2008)
Machias Seal Island (Gulf of Maine)	1998–2019	Survival age 0–1	0.30 (0.17–0.45)	This study
Machias Seal Island (Gulf of Maine)	1980–2003	Survival age 0–3	0.6895 (SE: 0.0189)	Breton, Diamond, and Kress (2006a)
Anda (Norway)	2005–2019	Survival, adult	0.84 (95% CRI: 0.79–0.88)	Reiertsen et al. (2021)
Eastern Egg Rock, Seal Island (Gulf of Maine)	1992–2003	Survival, adult	0.95 (SE: 0.01)	Breton, Diamond, and Kress (2005)
Fair Isle (Shetland)	1987–2013	Survival, adult	0.89 (SD: 0.05)	Miles et al. (2015)
Hornøya (Finnmark, Norway)	1990–2019	Survival, adult	0.85 (95% CRI: 0.76–0.91)	Reiertsen et al. (2021)
Isle of May (Scotland)	1984–2019	Survival, adult	0.90 (95% CRI: 0.85–0.94)	Reiertsen et al. (2021)
Machias Seal Island (Gulf of Maine)	1999–2011	Survival, adult	0.9238 (SE: 0.0089)	Breton and Diamond (2014)
Røst (Norway)	1990–2019	Survival, adult	0.90 (95% CRI: 0.87–0.93)	Reiertsen et al. (2021)
Runde (Norway)	2007–2019	Survival, adult	0.85 (95% CRI: 0.80–0.89)	Reiertsen et al. (2021)
Skomer Island (UK)	1970–2019	Survival, adult	0.9 (SD: 0.072)^e^	Newman et al. (2021)
Machias Seal Island (Gulf of Maine)	1998–2019	Survival, adult (4+)	0.85 (0.83–0.88)	This study
Machias Seal Island (Gulf of Maine)	1995–2019	Productivity	0.55 (0.49–0.61)	Major et al. (2021)
RAZO				
East coast of UK^a^	2014–2018	Productivity	0.564 (95% CI: 0.424–0.637)^b^	Searle et al. (2022)
Hornøya (Finnmark, Norway)	1989–2017	Productivity	0.68 (0.07–0.88)^f^	Jørgensen (2022)
Isle of May (Scotland)	1984–2009	Productivity	0.67 (95% CRI: 0.63–0.71)^g^	Lahoz‐Monfort et al. (2017)
Machias Seal Island (Gulf of Maine)	1998–2019	Productivity	0.53 (0.50–0.67)	This study
Machias Seal Island (Gulf of Maine)	1995–2019	Productivity	0.54 (0.50–0.58)	Major et al. (2021)
Matinicus Rock (Gulf of Maine)	2004–2009	Productivity	0.48 (0.35–0.61)	Kauffman (2014)
Shiant Islands (Outer Hebrides)	1970–1978	Survival age 0–1	0.16	Steventon (1982)
Machias Seal Island (Gulf of Maine)	1998–2019	Survival age 0–1	0.33 (0.22–0.45)	This study
Gannet Island (Labrador)	1996–2006	Survival age 0–2	0.482 (SE: 0.033)	Lavers et al. (2008)
Machias Seal Island (Gulf of Maine)	1999–2006	Survival age 0–2	0.778 (SE: 0.041)	Lavers et al. (2008)
Gannet Island (Labrador)	1996–2006	Survival age 3–11	0.970 (SE: 0.030)	Lavers et al. (2008)
Machias Seal Island (Gulf of Maine)	1999–2006	Survival age 3–8	0.912 (SE: 0.028)	Lavers et al. (2008)
Hornøya (Finnmark, Norway)	1995–2003	Survival, adult	0.919 (S: 2.5, tau: 0)^h^	Sandvik et al. (2005)
Isle of May (Scotland)	1984–2017	Survival, adult	0.923 (95% CRI: 0.897–0.945)	St. John Glew et al. (2019)
Skomer Island (UK)	1970–2019	Survival, adult	0.91 (SD: 0.048)^e^	Newman et al. (2021)
Machias Seal Island (Gulf of Maine)	1998–2019	Survival, adult (4+)	0.85 (0.81–0.89)	This study

^a^ Authors considered colonies within the region from Kent to Caithness, together with Orkney and Shetland.

^b^ Breeding success = # chicks fledged from # of nests.

^c^ Breeding success = chicks fledged per egg.

^d^ Median plus range (temporal); breeding success differed between the colonies and was calculated as chicks fledged per egg laid on the Isle of May and Hornøya, and as chicks fledged per egg hatched on Røst.

^e^ Temporal standard deviation.

^f^ Mean plus temporal range; breeding success = # of chicks that make it to 20 days from number of eggs laid.

^g^ Median plus 95% CRI, annual estimates ranged from about 0.56 (0.49–0.62) to 0.82 (0.75–87); breeding success = chicks that fledge from pairs that make attempt to breed.

^h^ S = square root of the total variance; tau = square root of the temporal variance.

### Availability, Observation, and Detection Figures

**TABLE A3:** Mean and range (where applicable) of estimates for both ATPU and RAZO of availability for resighting at time *t* given absent at time *t*−1 ($\gamma_{1}$) and present at time *t*−1 ($\gamma_{2}$), expected number of encounters per individual for pre‐breeding individuals (ages 0–3; ${ɛ}_{1}$) and breeding‐aged individuals (ages 4+; ${ɛ}_{2}$), and detection probability for pre‐breeding individuals (ages 0–3; $\rho_{1}$) and breeding‐aged individuals (ages 4+; $\rho_{2}$). Not that $\gamma_{1}$ cannot be estimated as all first‐year birds were available when they were first encountered. Range is not applicable for *γ* as these values were held constant across time in the model.

Species/Model parameter	Mean	Range^a^
ATPU		
$\gamma_{1}:2\mathrm{nd}\;\text{year}$	0.14 (0.12–0.15)	NA
$\gamma_{1}:3\mathrm{rd}\;\text{year}$	0.35 (0.33–0.38)	NA
$\gamma_{1}:4\mathrm{th}+\text{year}$	0.41 (0.40–0.42)	NA
$\gamma_{2}:1\mathrm{st}\;\text{year}$	0.01 (0.009–0.02)	NA
$\;\gamma_{2}:2\mathrm{nd}\;\text{year}$	0.27 (0.08–0.51)	NA
$\;\gamma_{2}:3\mathrm{rd}\;\text{year}$	0.48 (0.41–0.56)	NA
$\gamma_{2}:4\mathrm{th}+\text{year}$	0.62 (0.61–0.63)	NA
$\;{ɛ}_{1}$	1.07 (1.00–1.18)	0.91 (0.82–1.01) to 1.28 (1.16–1.42)
$\;{ɛ}_{2}$	1.63 (1.54–1.72)	1.37 (1.28–1.47) to 1.95 (1.84–2.05)
$\;\rho_{1}$	0.66 (0.58–0.74)	0.60 (0.56–0.64) to 0.72 (0.69–0.76)
$\;\rho_{2}$	0.80 (0.74–0.86)	0.75 (0.72–0.77) to 0.86 (0.84–0.87)
RAZO		
$\gamma_{1}:2\mathrm{nd}\;\text{year}$	0.13 (0.10–0.16)	NA
$\gamma_{1}:3\mathrm{rd}\;\text{year}$	0.39 (0.35–0.44)	NA
$\gamma_{1}:4\mathrm{th}+\text{year}$	0.42 (0.40–0.44)	NA
$\gamma_{2}:1\mathrm{st}\;\text{year}$	0.01 (0.006–0.03)	NA
$\gamma_{2}:2\mathrm{nd}\;\text{year}$	0.40 (0.07–0.81)	NA
$\gamma_{2}:3\mathrm{rd}\;\text{year}$	0.49 (0.33–0.64)	NA
$\gamma_{2}:4\mathrm{th}+\text{year}$	0.65 (0.63–0.67)	NA
$\;{ɛ}_{1}$	1.14 (1.01–1.38)	0.90 (0.72–1.12) to 1.91 (1.61–2.33)
${ɛ}_{2}$	1.62 (1.46–1.78)	1.27 (1.09–1.48) to 2.72 (2.51–2.94)
$\rho_{1}$	0.68 (0.55–0.85)	0.59 (0.51–0.68) to 0.85 (0.80–0.90)
$\;\rho_{2}$	0.80 (0.69–0.93)	0.72 (0.66–0.77) to 0.93 (0.92–0.95)

^a^ Upper and lower range of annual estimates given as mean and 95% CRI.

### tLTRE Proportional Contribution to Variance in Population Growth

**TABLE A4:** Mean proportional contribution (%), proportional contribution due to variance, and proportional contribution due to covariance of each demographic parameter to variance in realized population growth rate for ATPU and RAZ, where $\alpha_{2}$ = adult (after 1^st^ year) survival, $\alpha_{1}$ = first‐year survival, f = productivity, $N_{1}$ = # of 1^st^ year individuals, $N_{2}$ = # of 2^nd^ year individuals, $N_{3}$ = # of 3^rd^ year individuals, $N_{4}$ = #of 4^th^ + year individuals, and $N_{\mathrm{imm}}$ = # of immigrants. A negative contribution indicates that the demographic parameter reduces rather than adds to variance in population growth.

Species/Demographic parameter	Mean total contribution	Contribution due to variance	Contribution due to covariance
ATPU			
$\alpha_{2}$	50.510	44.169	6.341
$\;\alpha_{1}$	39.358	26.824	12.534
f	9.479	8.788	0.692
$\;N_{3}$	1.237	0.111	1.126
$\;N_{2}$	0.419	0.194	0.225
$\;N_{4}$	0.071	0.001	0.070
$\;N_{5}$	−0.071	0.001	−0.072
$\;N_{1}$	−1.003	0.268	−1.271
RAZO			
$\;\alpha_{2}$	73.887	66.253	7.634
$\;\alpha_{1}$	25.830	17.736	8.095
f	1.469	0.588	0.882
$\;N_{5}$	0.021	0.001	0.020
$\;N_{4}$	−0.021	0.001	−0.023
$\;N_{3}$	−0.088	0.024	−0.111
$\;N_{1}$	−0.407	0.076	−0.483
$\;N_{2}$	−0.693	0.040	−0.733
